# Re: Bilateral isolated epididymal agenesis in a 32 year old man

**DOI:** 10.1590/S1677-5538.IBJU.2015.0240

**Published:** 2015

**Authors:** Yadollah Ahmadi Asr Badr, Reza Sari Motlagh, Ehsan Sepehran

**Affiliations:** 1 Department of Urology, Tabriz University of Medical Sciences, Iran

*To the editor*,

In last issue of the Journal, Badr, et al (1) described a case report of 32 years old otherwise healthy man with bilateral epididymal agenesis. In medicine, agenesis refers to the failure of an organ to develop during embryonal time due to the absence of primordial tissue. Since secondary epididymal atrophy in this case could not be rolled out the term agenesis is inadequate and misleading. Particularly, because atrophic cauda epididymis of the left testis shown in the figure 2. Furthermore, cystic fibrosis carriers of one copy of the genetic mutation are usually healthy but may also be infertile due to undiagnosed secondary malformation of the Wolffian duct. Therefore, term epididymal atrophy instead agenesis would be more appropriate.

*Faruk Haziselimovic, MD* *Bahnhofplatz 11 4410 Liestal Liestal 4410* *Switzerland* *liestal@kindermedizin-zentrum.ch* *Fax: +41 619 279-099*
